# Mango: combining and analyzing heterogeneous biological networks

**DOI:** 10.1186/s13040-016-0105-5

**Published:** 2016-08-02

**Authors:** Jennifer Chang, Hyejin Cho, Hui-Hsien Chou

**Affiliations:** 1 Department of Genetics, Development and Cell Biology, Iowa State University, Iowa, 50011 Ames USA; 2 Department of Computer Science, Iowa State University, Iowa, 50011 Ames USA

**Keywords:** Systems biology, Heterogeneous data integration, Biological pathway analysis, 3D visualization, Graph mathematics

## Abstract

**Background:**

Heterogeneous biological data such as sequence matches, gene expression correlations, protein-protein interactions, and biochemical pathways can be merged and analyzed via graphs, or networks. Existing software for network analysis has limited scalability to large data sets or is only accessible to software developers as libraries. In addition, the polymorphic nature of the data sets requires a more standardized method for integration and exploration.

**Results:**

Mango facilitates large network analyses with its Graph Exploration Language, automatic graph attribute handling, and real-time 3-dimensional visualization. On a personal computer Mango can load, merge, and analyze networks with millions of links and can connect to online databases to fetch and merge biological pathways.

**Conclusions:**

Mango is written in C++ and runs on Mac OS, Windows, and Linux. The stand-alone distributions, including the Graph Exploration Language integrated development environment, are freely available for download from http://www.complex.iastate.edu/download/Mango. The Mango User Guide listing all features can be found at http://www.gitbook.com/book/j23414/mango-user-guide.

## Background

In the present Big Data era, one of the great challenges is to be able to compare or integrate diverse data types. Modern biological research produces large and heterogeneous data sets, and there are many ways to categorize or display each type of data. The 2014 Nucleic Acids Research Database Special Issue counted 1552 online biological databases [1]. It is often illuminating, even essential, to examine important biological problems using different types of data. For example, new discoveries often emerge when a biologist is able to interrogate gene expressions in the context of biological pathways [2]. A common method to analyze related data relies on graphs, or networks, where data of various types are linked and key network features or subsets are identified [3–5].

Many graph analysis solutions have been written in Java, most notably Cytoscape [6]. Started in 2002, Cytoscape has an impressive array of features. However, like other Java programs, the software slows to non-operational levels when handling large (>1 M link) biological networks due to Java Virtual Machine limitations [7]. Non-Java graph tools either do not provide analysis functions, or provide only libraries which users must incorporate into their own software solutions. Overall, many graph tools focus solely on one functionality, i.e., either analysis or visualization, and require users to integrate two or more tools for one project. Multi-graph comparison and integration are further complicated by differing graph attributes from heterogeneous data sets. Many tools ignore or limit the number of attributes associated with a graph. A comparison of currently available graph analysis and visualization software [6, 8–10] is given in Table 1.

**Table 1:** Comparison of graph visualization software

Software	Code	Graph analysis features	Visualization	Limitations
Cytoscape	Java	· Many algorithms for systems biology	· 2D predetermined layout	· Can only merge 2 graphs at a time
(v. 3.2.1)		· Can add GO or KEGG attributes	· 3D predetermined layout (via plug-in)	· 6 min to load a network with 4 M links
		· Plug-ins available	·	but no visual afterward
Gephi	Java	· Intuitive graph statistics	· 2D and 3D layouts but graphs cannot be	· Cannot display multiple graphs on one
(v. 0.8.2)		· Automated graph algorithm citation	rotated in 3D	screen
		· Generalized for all types of graphs	· Graph layout animation helps maintain	· Limited by JVM constraints; cannot load
		· Plug-ins available	mental map	a network with 4 M links
GUESS	Java	· GYTHON, a language for graph analysis	· 2D layout only	· Cannot be run on MacOS 10.9, Windows
		· Can map information attributes to visual	· Update with user commands	7, or Redhat Linux 6.0
		attributes		
Graphviz	C	·No graph analysis capabilities	·Rich set of predetermined 2D layouts	·Not an interactive system
			· Streamlined command line interface	· Cannot efficiently handle graphs over
				100 nodes
Neo4j	Java	·Graph database system	·Relies on JSON for visualization	·Designed as a backend to database sup-
(v. 2.1.7)		· Cypher graph query language	· 2D layouts only	port rather than for visualization
		· Queries are based on a combination of	· Have to click a node or link to see its	· Nodes are only labeled by numbers
		topology and attributes	attributes on a separate panel	· The whole database is one huge graph
Tulip	C++	· A set of C++ libraries for graph analysis	· 2D visualization	· More useful to users who program C++
(v. 4.6.1)		· Can also be run as stand-alone program	· 3D is available through plug in	or python directly
		· Plug-ins can be created in Python	· Had some 3D layout algorithms	· More analysis than visualization features
NetworkX	Python	· Python module for graph analysis	· Must export to other software or	· Useful only as an analysis tool
(v. 1.6.1)		· Rich set of network algorithms	modules for visualization	
Mango	C++	· Provides general graph mathematics	· Interactive 3D layouts and controls	· Does not yet have plug-in feature
(v. 1.10)		· Heterogeneous graph analysis with ease	· Real-time large graph visualization	· Does not yet use GPU speedup
		· Takes ∼30 s to load a 4M link network	· User customizable visual attributes	· Limited set of preset layouts

Benchmarks were performed on a 2010 Mac mini that has 8 Gb RAM and runs 64-bit MacOS X 10.9 with a 2.4 GHz Intel Core 2 Duo processor. All software were run using their default configurations

To address these limitations, we have developed a stand-alone graph analysis and visualization software environment called Mango to aid biologists and other researchers efficiently integrate and explore heterogeneous networks larger than previously possible. A 4 million link network can be loaded into Mango in 30 seconds on a Mid 2010 Mac mini computer with a 2.4 GHz (Gigahertz) Intel Core 2 Duo processor and 8 GB RAM (random access memory). As a comparison, Cytoscape took 6 minutes to load that same network file on the same computer using its default configurations. Mango possesses the scalability to handle larger networks, the expressive power of a new Graph Exploration Language (Gel) and the convenience of unlimited graph attributes with automatic graph attribute merging and promotion. Within the integrated development environment, Gel commands can be edited, run line-by-line, or saved as scripts to reproduce results. Script files enhance the speed and reproducability of analysis [11]. Mango provides both comprehensive graph analyses and real-time 3-dimensional (3D) visualization. Mango is a cross-platform C++ program that runs on Mac OS X 10.9 or later, Windows 7 or later, and many Linux variants. It is freely available from our website (http://www.complex.iastate.edu/download/Mango) and the Mango User Guide is hosted at GitBook (http://www.gitbook.com/book/j23414/mango-user-guide).

## Implementation

### The Mango user interface

**Fig. 1:**
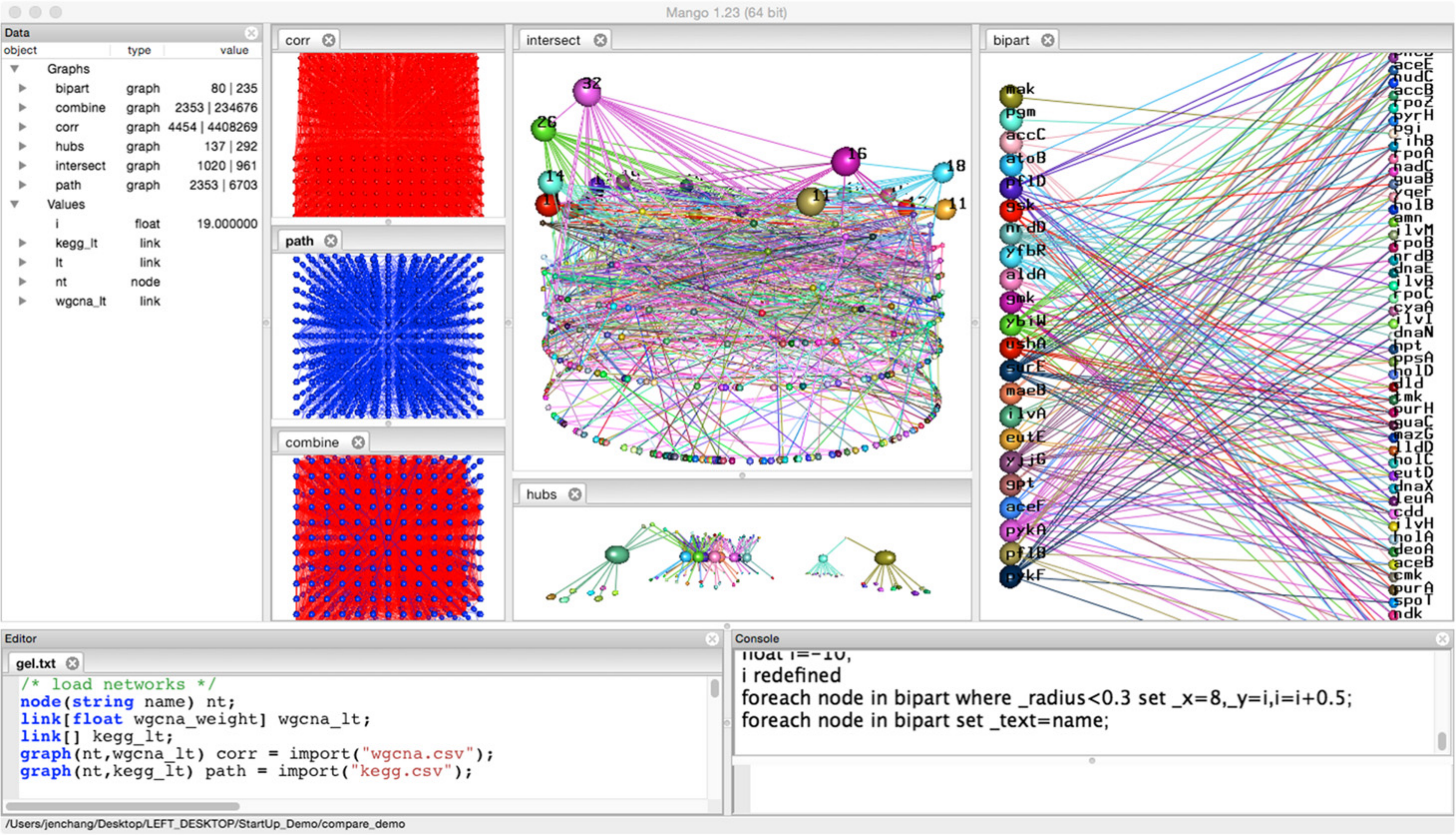
Mango user interface. The main window is divided into four areas: data list (left), graph canvases (middle 3D visualizations), Gel editor (*bottom left*), and Gel command console (*bottom right*). Shown in the graph canvas area are the following networks: Left column: WGCNA correlation network, KEGG biological pathway network and their combined networks; Middle column: crown-plot of the intersection network between correlation and pathway networks and extracted hub genes sub-network; and Right column: hub and in-betweener genes laid out in a bipartite graph where nodes are labeled by gene names

**Fig. 2:**
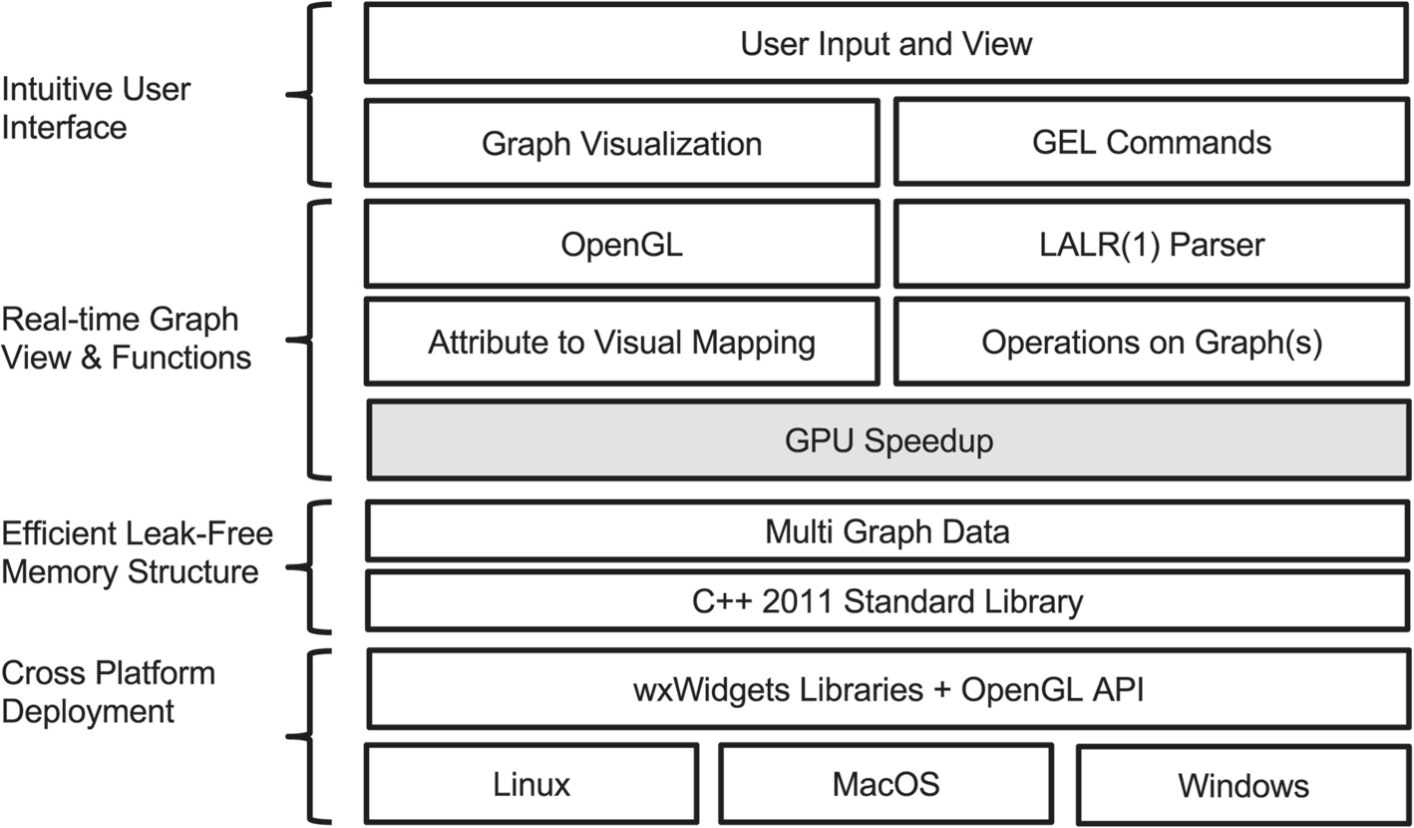
System architecture. The Mango software is made up of multiple code layers seamlessly stacked up to form the stand-alone program. The GPU speedup layer is not included in some Mango versions

Mango updates its display in real-time at each stage of analysis to facilitate the integration and modification of multiple large networks. Mango contains a primary window divided into four areas (Fig. 1). The graph canvas area is fully interactive, responding to mouse and keyboard actions to zoom, move, rotate, and auto-layout the displayed graphs. By dragging and rearranging tabs, multiple graphs can be viewed simultaneously, easing multi-network comparison. Mango functions are mostly carried out through its command console or Gel code editor. The Gel code editor allows commands to be run line-by-line, edited, and saved as Gel script files. Gel script files can then be shared among researchers, reproducing a 3D layout or network analysis pipeline. Finally, the data area lists currently loaded graphs, their sizes and attributes. Interactive real-time network visualization in Mango helps hone and refine each step of analyses. Mango is built on multiple layers of implementation that are seamlessly combined to form an integrated solution for graph analysis (Fig. 2).

### The Graph Exploration Language (Gel)

A graph is defined as a set of nodes (*V*) and links (*E*) where a node represents some entity and a link represents a relationship between a pair of entities. In practice, graphs also have added annotations called attributes. Currently, Gel provides four basic data primitives *string*, *int*, *float* and *double* as well as aggregate data types *node* (*V*_*attr*_), *link* (*E*_*attr*_) and *graph*.

$$\begin{array}{*{20}l} G=\{V,E\} && \text{where} && V=\{v_{1},v_{2},v_{3},...,v_{n}\}\\ && && E \subseteq \{(v_{i},v_{j})|v_{i},v_{j} \in V\} \end{array} $$

$$\begin{array}{*{20}l} V_{attr} &=\{a_{1},a_{2},a_{3},...,a_{m_{1}} | type(a_{i}) \in \{int, float, double, string\}\} \\ E_{attr} &=\{a_{1},a_{2},a_{3},...,a_{m_{2}} | type(a_{i}) \in \{int, float, double, string\}\} \end{array} $$

**Fig. 3:**
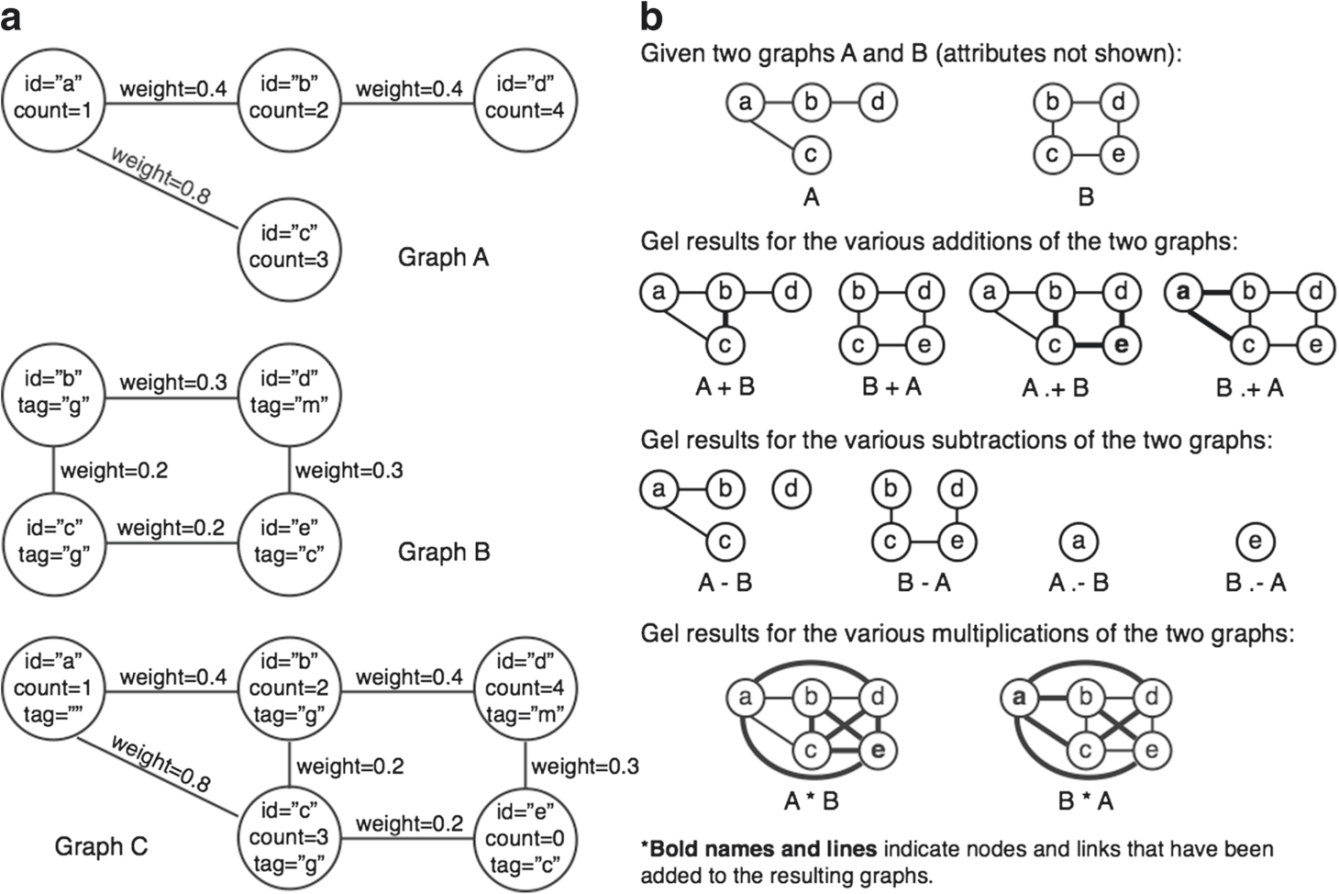
Graph Exploration Language examples. **a** Graphs A and B have different node attributes. Graph C is the result of attribute merging and promotion of A and B. **b** Graph mathematics. Given two graphs A and B, the dotted addition A.+ B combines nodes and links from graph A and graph B. The non-dotted addition A + B combines graph A with links of Graph B whose end nodes are already contained in graph A. Graph subtraction works similarly. Graph mathematic results depend on operand order; attribute merging and promotion are handled automatically as described in the main text but are not shown in this figure



Each nodes and link type can have any number of attributes of the four primitive types in any order, and each of the attributes has a distinct name and specified data type (e.g. *string*, *int*, *float*, and *double*). The first attribute in a node type must be a *string* to denote the node name, and a link is identified by a pair of node names. All node and link attributes have default values, which are usually zero for numeric types or the empty string, but users can define other default values during node and link type declarations. Graphs are defined based on a pair of *node* and *link* types. For example, the following Gel code defines and initializes two graphs *G*_*A*_ and *G*_*B*_, also shown in Fig. 3a. Node type and link type are defined with the given attributes inside parentheses and brackets; the brackets denote non-directional link types (whereas arrows <> denote directional link types). For example, *G*_*A*_ is declared with *ntA* and *ltA*, and is also initialized by the graph literals enclosed within the braces.

Other than defining a graph in the native graph exploration language, Mango can read graph data in tabular or CSV (comma separated values) format using the **import** command. A properly formatted graph file lists nodes with their attributes and then links with their attributes. A single line containing a hyphen separates the node list from the link list. The full description of the **import** command is in the Mango User Guide.

Mango system-defined graph attributes are appended to user defined attributes. The system-defined attributes are related to the 3D visualization of a network and define such attributes like node position, node color, or link width. Therefore, generating any 3D visualization is a matter of mapping user defined information attributes to system defined visualization attributes [12]. By dynamically changing these mappings, animations and simulations can be accomplished in Mango. A full listing of the visualization attributes is in the Mango User Guide.

### Standards for combining heterogeneous graphs

When combining two or more graphs, much of the confusion stems from what will happen to the nodes and links. Since a graph contains both node and link sets, our formally defined dotted and non-dotted graph mathematic operators allow users to specify node-centric or link-centric operations precisely. Recall the two graphs *G*_*A*_ and *G*_*B*_.

$$\begin{array}{*{20}l} G_{A}=\{V_{A},E_{A}\} && G_{B}=\{V_{B},E_{B}\} \end{array} $$

Merging nodes and links is represented by the dotted addition.

$$\begin{array}{*{20}l} G_{A}\, . +G_{B} &= \{V_{A} \cup V_{B}, E_{A} \cup E_{B}\} \end{array} $$

However, suppose that the user is only concerned with the nodes in *G*_*A*_, such as a set of important genes, and merely wants to combine the new links between those genes from *G*_*B*_. The non-dotted addition merges links from *G*_*B*_ only between nodes already in *G*_*A*_.

$$\begin{array}{*{20}l} G_{A} + G_{B} &= \{V_{A}, E_{A} \cup \{(v_{i},v_{j})|v_{i},v_{j} \in V_{A}, (v_{i},v_{j})\in E_{B}\}\} \end{array} $$

In a similar fashion, dotted and non-dotted subtraction between two graphs are defined as follows.

$$\begin{array}{*{20}l} G_{A}\, .- G_{B} &= \{V_{A} \setminus V_{B}, (v_{i},v_{j})|(v_{i},v_{j}) \in \{E_{A} \setminus E_{B}\}, v_{i}, v_{j} \in \{V_{A}\setminus V_{B}\}\}\\ G_{A} - G_{B} &= \{V_{A}, E_{A} \setminus E_{B}\} \end{array} $$

Other operations such as producing intersections and bipartite graphs are defined as follows.

$$\begin{array}{*{20}l} G_{A}\, .\&\ G_{B}&=\{V_{A}\cap V_{A}, E_{A} \cap E_{B}\}\\ G_{A}\ \&\ G_{B}&=\{V_{A}, E_{A} \cap E_{B}\}\\ G_{A}\ *\ G_{B} &= \{V_{A} \cup V_{B}, E_{A} \cup E_{B} \cup \{(v_{i},v_{j})|v_{i} \in V_{A}, v_{j} \in V_{B}, v_{i} \neq v_{j} \}\}\\ G_{A}\ **\ G_{B} &= \{V_{A} \cup V_{B}, E_{A} \cup E_{B} \cup \{(v_{i},v_{j})|v_{i} \in V_{A}, v_{j} \in V_{B}\}\} \end{array} $$

The above mathematics can be extended across multiple graphs to create unions (*G*_*A*_.+*G*_*B*_.+*G*_*C*_), differences (*G*_*A*_.−*G*_*B*_.−*G*_*C*_ or *G*_*A*_−*G*_*B*_−*G*_*C*_), intersections (*G*_*A*_.& *G*_*B*_.& *G*_*C*_) and inverse graphs (*G*_*A*_∗*G*_*A*_−*G*_*A*_). The graph operations can be mixed and matched to produce more complex results. Figure 3b demonstrates a few of the graph mathematics visually.

When graphs are combined in mathematical operations, attributes from two graphs might conflict. For example, the link between *b* and *d* nodes in *G*_*A*_ may have a *weight* attribute of 0.4 while the link between *b* and *d* nodes in *G*_*B*_ may have a *weight* attribute of 0.3. Gel handles attribute conflicts by giving preference to the left operand. During the operation *G*_*A*_.+*G*_*B*_, the left operand *G*_*A*_ takes precedence and the resulting graph will have *weight* value 0.4. An exception to this rule is when the conflicting attributes in *G*_*A*_ happen to be at their default values (default values can be defined by users). In those cases, the attributes of graph *G*_*B*_ will be copied. This automatically merges useful non-default information from *G*_*B*_ into the resulting graph.

When heterogeneous graphs are combined, their unique attributes can be selectively preserved. Recall that the nodes in *G*_*A*_ have attributes *id* and *count* while nodes in *G*_*B*_ have attributes *id* and *tag*.

$$\begin{array}{*{20}l} V_{A,attr}&=\{id, count\} && E_{A,attr}=\{weight\}\\ V_{B,attr}&=\{id, tag\} && E_{B,attr}=\{weight\} \end{array} $$

Because nodes in *G*_*B*_ only share the *id* attribute with *G*_*A*_, when *G*_*B*_ is added to *G*_*A*_ as in *G*_*A*_.+*G*_*B*_, the *count* attribute of nodes copied from *G*_*B*_ is automatically set to the default value 0 but their *tag* attribute is ignored. To preserve both *G*_*A*_ and *G*_*B*_ attributes, users can define a new node type that includes all attributes. This is called attribute promotion. In our example, a new node type containing *id*, *count* and *tag* attributes is defined and used by the new *G*_*C*_ to receive all attributes from *G*_*A*_ and *G*_*B*_.

$$\begin{array}{*{20}l} V_{C,attr}=&\{id, count,tag\} && E_{C,attr}=\{weight\} \end{array} $$

However, simply writing *G*_*C*_=*G*_*A*_.+*G*_*B*_ will not work as the *tag* attribute from *G*_*B*_ is already lost after the addition of *G*_*B*_ to *G*_*A*_ but before the result is assigned to *G*_*C*_. The correct steps to preserve graph attributes during heterogeneous graph mathematics are demonstrated below (Fig. 3a):



Flexible node and link type definition coupled with an intuitive set of attribute promotion and merging rules ease the combination of heterogeneous graphs in Gel. Thus users can focus on graph level operations instead of attribute level selection, sorting, and merging.

Many graph analyses require traversing all nodes and links to perform a calculation based on graph attributes or topology. Gel provides the *select* command to pull out a subgraph based on user-defined conditions. These conditions can be related to stored attribute values or topology properties. Gel also allows mapping or computing new attribute values across a graph on a per-node or per-link basis with the *foreach* command, which efficiently applies a set of user-defined calculations across all nodes or links that optionally meet certain conditions. The same command can also be used to tally attribute values across all nodes and links. The following demonstrates the two types of Gel commands:



In addition to the data types, graph mathematics, automatic attribute handling and traversal commands; Gel also provides commands for object modification, data examination, input and output, code execution, graph construction, and simulation. A growing set of built-in functions for mathematics, visualization control, graph layouts, and statistical reporting are also provided. To explore all Gel commands and functions, type the help command in Mango or consult the online User Guide.

The Mango system and its Graph Exploration Language are data agnostic, meaning that any type of network can be loaded and analyzed – users have total control of node and link attribute definitions and their associations within Mango. Our goal is to make this software widely available to all researchers and promote its use in solving ever more complex biological research problems.

### KEGG connect

**Fig. 4:**
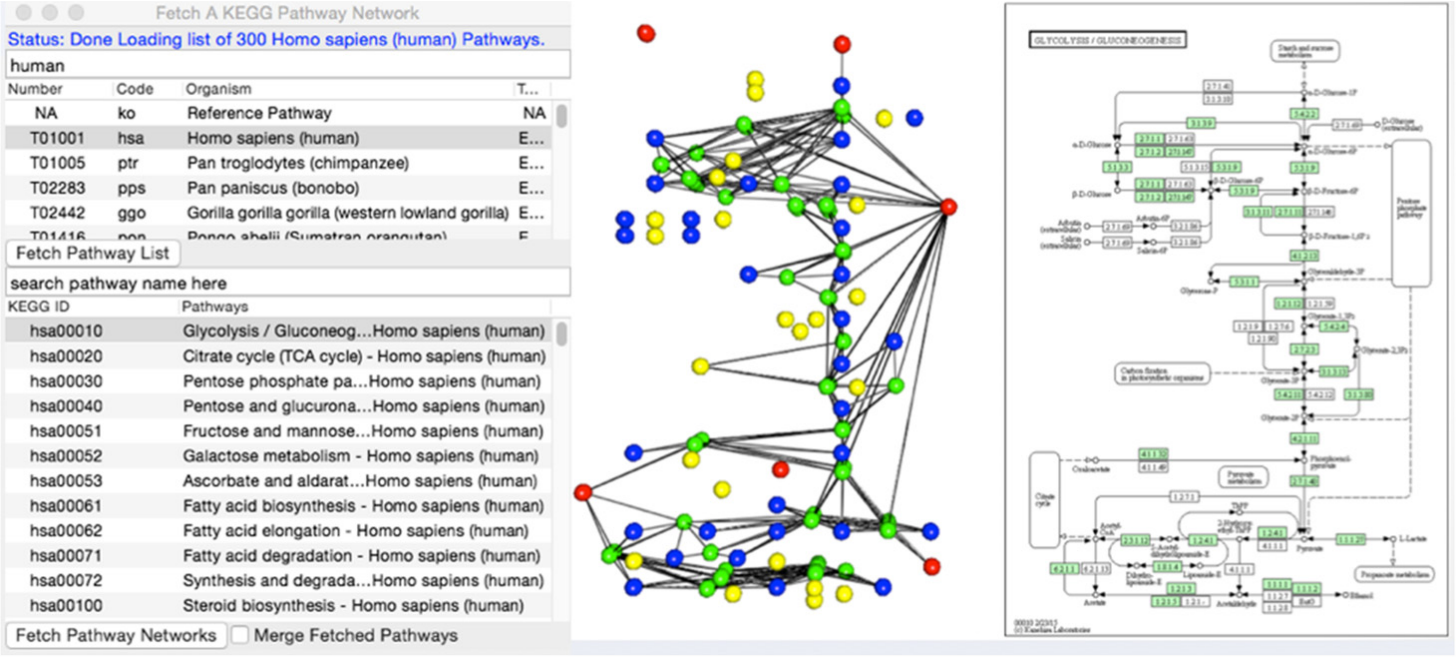
KEGG Connect. (*Left*) The KEGG Connect dialog lists currently available organisms and pathways in the KEGG database. Users can fetch multiple pathways individually or merge them into one network by checking the “Merge Fetched Pathways” box. (*Middle*) Mango maintains the x-y coordinates from KEGG website drawing and colors nodes red (pathway map), *green* (enzymes), *blue* (compounds), and *yellow* (orthologs). (*Right*) Corresponding KEGG website drawing for the same pathway

The KEGG Connect dialog demonstrates how Mango can fetch network data directly from online biological databases. KEGG Connect queries the KEGG (Kyoto Encyclopedia of Genes and Genomes) database (http://www.genome.jp/kegg) and selectively downloads pathways grouped by organisms. Within the downloaded pathway, nodes maintain their 2-dimensional (2D) coordinates from the KEGG visualization. The nodes are colored red, blue, green and yellow representing pathway maps, compounds, genes, and orthologs respectively (Fig. 4). Multiple pathways can be downloaded either as individual networks or as one merged network. If multiple networks are merged, each pathway will be given a different z coordinate value, so the pathways are layered in 3D space. We intend to connect Mango to more biological databases soon.

## Results and discussion

We present a few network analysis examples to illustrate the use of Mango in this section. Examples of comparing different types of biological networks and the scalability of Mango to large networks are provided.

### Network data collection

Four large *E. coli* network data sets were collected. The *corr* 4 M link network was computed using the WGCNA (weighted gene coexpression network analysis) package in R [13] on microarray data measuring the expression of 4454 *E. coli* genes in cells grown under 10 different conditions (GSE61736, [14]). The *path* biological pathways of *E. coli* were downloaded from the KEGG database (http://www.genome.jp/kegg) and combined into a single pathway network. The *go* network was constructed using *E. coli* GO (gene ontology) information retrieved from the gene ontology website (http://geneontology.org/page/download-annotations); *E. coli* genes that share at least one GO term are linked. Finally, the protein-protein interaction (*ppi*) network was retrieved from the supplementary materials of a 2014 paper [15]. Sizes and attributes for the 4 large networks are summarized in Table 2.

**Table 2:** Summary of 4 large heterogeneous biological networks for *E. coli*

Network	Nodes	Links	Node attribute(s)	Link attribute(s)
corr	4,454	4,408,269	gene name	WGCNA correlation weight
path	2,353	6,703	gene name	none
go	3,764	2,208,090	gene name	count and string of shared GO terms
ppi	2,042	3,888	gene name	source of evidence (Y2H, LIT or both)

Unconnected nodes and duplicate links have been removed from some of the networks. In all 4 networks, nodes are identified by gene names and differ in their link attributes

### Large heterogeneous network comparison

For all networks, nodes are identified by gene names with no additional attributes, thus the following node type declaration can be shared among the networks:



All networks have undirected links but differ in their link attributes (the path network does not contain any link attributes), thus the following 4 link type declarations are used to load the different networks:



After the node and link type declarations, the *corr* network, *path* network, *go* network, and *ppi* network can be imported into Mango for all-to-all network comparisons:



For the integration of the networks, a common link type including all available link attributes is declared:



Once the networks are loaded into Mango, Gel mathematics allow network integration and comparisons. For example, the comparison of the *corr* and *path* networks are visualized in the top two panels in the left column of Fig. 1. The top middle panel in Fig. 1 is the result of the following Gel intersect operation.



**Fig. 5:**
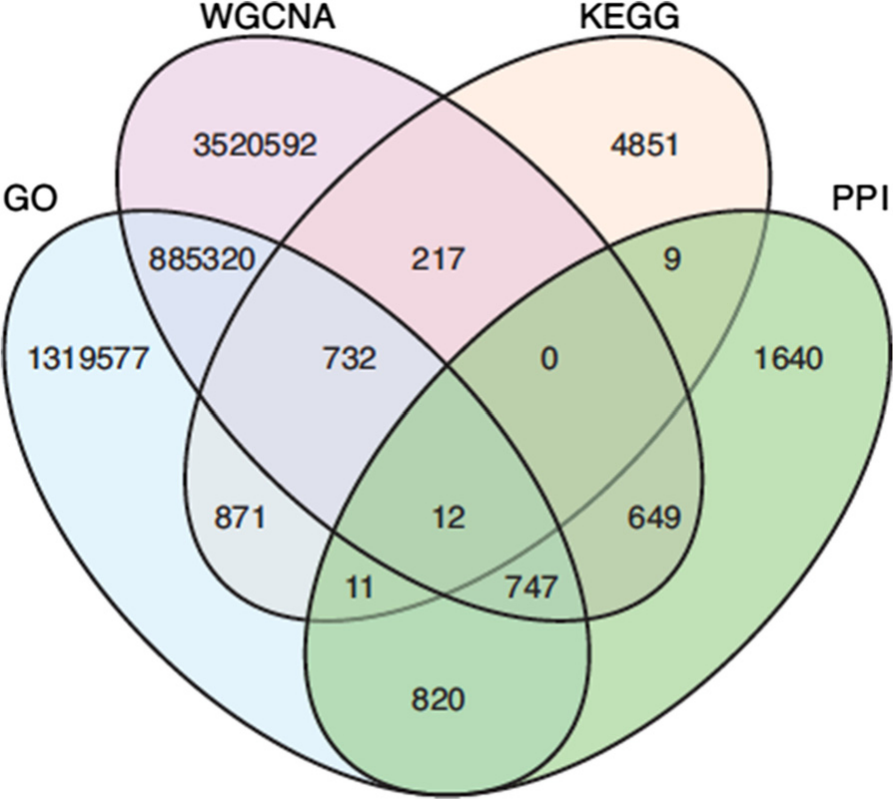
Biological network comparisons. Link intersections among the *corr*, *path*, *go* and *ppi* networks. The intersections were worked out using Gel commands. WGCNA is the gene-to-gene correlation network *corr* computed from *E. coli* microarray data. PPI is the protein-protein interaction network *ppi* of *E. coli*. GO is the network *go* that connects any two *E. coli* genes sharing at least one gene ontology term. KEGG is the entire KEGG biological pathway network *path* of *E. coli*

The *corr-path* intersection network contains 961 links with 1020 nodes. The all to all comparisons of these four networks were completed in Mango and the common links among the networks were summarized in Fig. 5. All possible intersections among the four *E. coli* networks can be worked out with a few lines of Gel code each. Bench-marked time for different types of Gel mathematics between the large *corr* and *path* networks are listed in Table 3.

**Table 3:** Benchmarking the speed of Gel mathematics on massive graphs

Gel operation.	Time (in seconds)	Average
4 *M*+=8 *K*	0.92, 0.35, 0.27, 0.60, 0.56	0.54
8 *K*+=4 *M*	1.25, 1.15, 1.03, 1.02, 1.02	1.09
4 *M*−=8 *K*	0.52, 0.33, 0.62, 0.33, 0.25	0.41
8 *K*−=4 *M*	1.09, 1.28, 1.09, 1.16, 1.19	1.16
4 *M*.+=8 *K*	0.69, 0.60, 0.57, 0.31, 0.40	0.51
8 *K*.+=4 *M*	12.06, 12.09, 12.05, 12.23, 12.32	12.15
4 *M*.−=8 *K*	0.55, 0.41, 0.25, 0.26, 0.32	0.36
8 *K*.−=4 *M*	0.90, 0.85, 0.83, 0.98, 0.74	0.86
4 *M*∗=8 *K*	22.94, 23.74, 23.35, 22.98, 23.03	23.21
8 *K*∗=4 *M*	36.75, 35.33, 35.23, 35.38	35.67
*c* *o* *p* *y*=4 *M*	7.90, 7.76, 7.85, 7.73, 7.87	7.82
*c* *o* *p* *y*=8 *K*	0.30, 0.52, 0.45, 0.34, 0.29	0.38

The 4 M link network is the gene correlation network generated by WGCNA. The 8 K link network is the combined KEGG pathway network. Benchmarks were performed consecutively on a 2010 Mac mini that has 8 Gb and runs 64-bit MacOS X 10.10 with a 2.4 GHz Intel Core 2 Duo processor. The time to copy the networks is also listed. All operations, including the copy operation, were performed using single thread in RAM

### Flexible real-time network exploration and visualization

Over-plotting of nodes and links becomes more of a challenge as network sizes get bigger. For example, the *corr* and *path* networks and their combination can be visualized in Mango but provide limited biological interpretation (the left column of panels in Fig. 1). In this example, we continue to explore the intersection of the two networks by querying certain node and link attributes, imposing thresholds to reveal important features, and map these features to network visualization.

First we arrange all nodes in the intersection network along a circle in the x-y plane and map the node connectivity to their z-axis coordinates. Nodes are assigned random colors and higher z-axis node colors are bled down the links to emphasize hubs. Nodes above a threshold are emphasized by increasing their radius and labeling them with gene names and connectivity.



The resulting network layout, called a **crown-plot**, is shown on the top pane in the middle column of Fig. 1. The hub genes and their links can be pulled into a new sub-network. The sub-network called hubs is then flattened and spread out using a force-directed layout built into the graph panel by right-clicking on the panel. The hub genes are raised one level. Genes that are not themselves hubs but connect two or more hubs are raised to a third level. The following Gel code accomplishes all these except the force-directed layout, which is performed by right-clicking on the panel:



The 3-layer hubs network is shown in the lower panel in the middle column of Fig. 1, which contains other genes on the bottom layer, hub genes on the middle layer and in-betweener genes on the top layer. It is worth mentioning that the in-betweener genes on layer 3 would have been obscured by other genes in a simple list of genes ordered by connectivity. We can further pull out the hubs and in-betweeners into another sub-network for closer inspection with the following Gel code:



This sub-network is laid out as a bipartite graph shown on the right panel in Fig. 1, with hubs on the left and the in-betweeners on the right. This example shows how to map informational attributes of a graph to its visual attributes using Mango. The resulting visual displays help the user decide threshold values, extract sub-networks of interest, and further explore the data.

### Microarray expression combined with KEGG biological pathways

*E. coli* gene expression under control and multiple treatment conditions were measured by microarrays (GSE61736, [14]). A subset of the data containing one control and one treatment expression values was loaded into Mango and overlaid onto downloaded *E. coli* KEGG biological pathways. The expression data, *E. coli* KEGG pathways, and Gel script are available for download from https://github.com/j23414/Mango_Workshop.

**Fig. 6:**
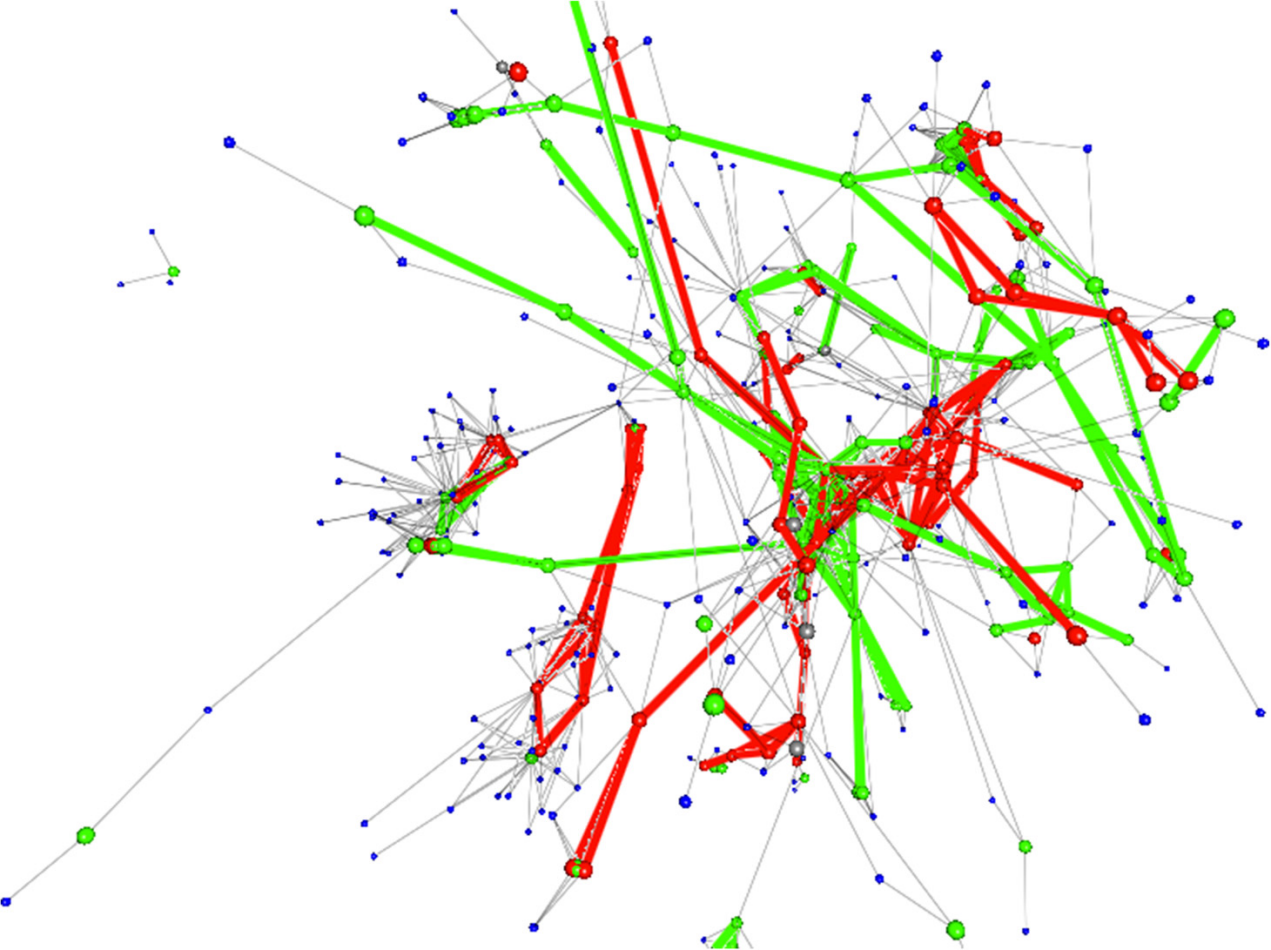
Gene expression combine with KEGG. A 3D KEGG network visualization comparing the *E. coli* gene expression values obtained under a treatment condition and a control condition. In addition to coloring and resizing the genes (i.e., node) of the network based on expression changes related to the control, pathway links are also highlighted in green or red depending on up or down expressed genes they connect in a pathway. The highlighted links allow a whole pathway to be easily discerned as up or down regulated



The results of the visualization can be seen in Fig. 6. Genes are colored green or red where their expression levels are up or down relative to the control condition. KEGG pathway components that do not have mapped gene expression values are colored gray. Compounds are colored blue and are largely ignored although they could be used to incorporate metabolomic concentration values. The Gel commands to color gene nodes are given below:

More than coloring nodes in a network, we are able to color the links and thereby highlight entire pathways that are up or down-regulated. This is possible because KEGG pathways also contain gene to gene links, not just gene to compound links.



The final network can be saved and reloaded to regenerate the same 3D visualization.



Mango networks are saved natively into Gel commands, thus running the saved code recreates the original graphs in Mango. In addition, the networks can be exported to tabular data using the **export** command. The tabular data can then be read by many other software programs, e.g., Excel, R, Matlab, Cytoscape, and other graph software or databases. Full descriptions of the interoperability and other features of Mango are available in the User Guide.

## Conclusion

We have developed a powerful new program Mango for multi-network analysis and visualization. Mango enables scientists to test hypotheses on large heterogeneous networks, identify crucial features, and extract analysis results all within its integrated environment. Compared with existing programs, Mango extends the capability and convenience of large heterogeneous data analysis on a personal computer.

The Mango system was designed to be data agnostic, meaning that any type of network data can be loaded and analyzed – users have total control on node and link attribute definitions and their associations within Mango. Mango can load networks with millions of links, integrate and explore large amounts of data following Gel commands, and help users deduce predictions or outcomes that can be validated in labs. It is our goal to make this software widely available to all researchers to promote its use in solving ever more complex biological research problems. As Mango developers, we will continue to provide support and further develop the software according to user needs.

## Availability and requirements

- **Project name:** Mango 1.24.
- **Project home page:**http://www.complex.iastate.edu/download/Mango/
- **Operating system(s):** Mac OS X 10.9 or later, Windows 7 or later, and Linux variants. Both 32- and 64-bit operating systems are supported.
- **Programming language:** C++
- **Other requirements:** An Internet connection for online database access.
- **License:** Free versions available; specific license agreement included with each distribution.
- **Any restriction to use by non-academics:** Specific restrictions included with each distribution and license agreement.

## Abbreviations

2D, 2-dimensional; 3D, 3-dimensional; CSV, comma separated values; Gel, graph exploration language; GO, gene ontology; GHz, Gigahertz; KEGG, Kyoto Encyclopedia of Genes and Genomes; PPI, protein-protein interaction; RAM, random access memory; WGCNA, weighted gene correlation network analysis
